# This *i*s what life with cancer looks like: exploring experiences of adolescent and young adults with cancer using two visual approaches

**DOI:** 10.1007/s00520-021-06775-9

**Published:** 2022-01-05

**Authors:** Zarah M. Bood, Floor van Liemt, Mirjam A. G. Sprangers, Annita Kobes, Yvonne Weeseman, Michael Scherer-Rath, Jacqueline M. Tromp, Hanneke W. M. van Laarhoven, Esther Helmich

**Affiliations:** 1 Department of Medical Oncology, Cancer Center Amsterdam, Amsterdam University Medical Centers, University of Amsterdam, VU Medical Center, De Boelelaan 1117, 1081 HV Amsterdam, The Netherlands; 2 Stichting F|Fort Foundation, Haarlem, The Netherlands; 3 Department of Medical Psychology, Amsterdam University Medical Centers, University of Amsterdam, Amsterdam, The Netherlands; 4 Faculty of Philosophy, Theology, and Religious Studies, Radboud University–Nijmegen, Nijmegen, The Netherlands; 5 Amsta Healthcare Organisation, Amsterdam, the Netherlands

**Keywords:** Cancer, AYAs, Patients’ experiences, Visual tool, Rich pictures, Photovoice

## Abstract

**Introduction:**

Talking about illness experience can be challenging for adolescents and young adults (AYAs) with cancer. Visual tools, in addition to spoken language, might make this easier, such as rich pictures and photovoice. We aimed to obtain a comprehensive view of the cancer experience of AYAs by using rich pictures and photovoice.

**Methods:**

AYAs (18–35 years old) who had any type of cancer, or were in remission from cancer, were eligible. AYAs drew rich pictures about their experience of living with cancer and explained these during subsequent interviews. Some of the AYAs also participated in photovoice and spent two days with a photographer to make photos about their illness experience. Rich pictures and photos were first analyzed separately, using open coding, after which the identified themes were compared.

**Results:**

Twelve AYAs made rich pictures (RPs), of whom seven also participated in photovoice. The two most predominant themes emerging from the data were struggles related to the future and defining one’s identity. The AYAs expressed concerns for the future related to relationships, education, and employment. Relating to defining one’s identity, many AYAs expressed that the cancer had a negative impact on their body- and self-image. The main themes were visible in the RPs as well as in the photovoice; however, subtle differences in sub-themes were found.

**Conclusions:**

We found that cancer has an effect on many aspects of AYAs’ lives. Further research on how the identified themes play a role in the lives of AYAs with cancer is needed.

## Introduction

Receiving a diagnosis of cancer is life altering at any age, but especially for adolescents and young adults (AYAs), it can be very disruptive. The life of AYAs, at the age from 18 to 35, is often marked by developmental milestones, including transitioning into further education or employment, leaving childhood homes, gaining financial independence, and forming intimate emotional and sexual relationships [1–3]. Therefore AYAs represent a unique patient population, with needs and challenges that differ from those of adults and children with cancer [1–4]. Nevertheless, AYAs with cancer have been an understudied population and their experiences have rarely been investigated. Consequently, there is a lack of interventions that meet their needs [1, 3]. If we could obtain a comprehensive view of the impact of a cancer diagnosis at such an age, further research might be able to design interventions to support AYAs and assess their effects.

One of the difficulties that can be encountered when exploring cancer experiences of patients is that words may be insufficient. Thus, questionnaires or interviews possibly give insight into a limited part of AYAs cancer experiences [5–9]. Alternatively, AYAs may be invited to tell their story through visual methods, in addition to spoken language [8–12]. The combination of visual and verbal methods, such as drawings and comics, was found to be helpful in uncovering experiences of children and adults with cancer [12–17]. Although it has been suggested that AYAs may also benefit from sharing their experiences in a visual way, few studies actually attempted to investigate this [1, 18].

A visual tool that could possibly provide a comprehensive view of the experiences of AYAs with cancer is rich pictures (RPs) [19]. RPs are drawings that one creates to express one’s experiences, which may encompass different elements, e.g., people, interactions, and feelings [9–11, 20, 21]. We recently conducted a study with a group of adults (mean age 62) with advanced cancer who were asked to draw an RP about their experience of living with cancer [19, 22]. The findings suggest that RPs can help patients to tell their story about how cancer affects their lives. The RPs can provide insight into what is most important to the patient and may shed light on how multiple aspects of well-being, such as physical, emotional, and social well-being, are affected by cancer [19, 22]. Nonetheless, RP research has not yet been performed amongst AYAs with cancer.

Another upcoming visual tool to explore cancer experiences is photovoice [23, 24]. In photovoice, participants are invited to document meaningful aspects of their experience through photographs [23]. The photovoice methodology has not been used widely amongst cancer populations [24]. Some studies, such as Wong et al. [23] and Georgievski et al. [25], have adopted photovoice to explore the experiences of, respectively, older adults or teenagers (under 18 years) with cancer. These studies have demonstrated that photovoice can create a valuable opportunity for patients with cancer to share their experiences with others [24]. However, when focusing on photovoice research amongst AYAs, there are limited studies and the few available have included cancer survivors only, not AYAs currently living through cancer [24]. There are no studies that have adopted photovoice with AYAs in active cancer treatment, even though experiences between AYAs with active cancer treatment and cancer survivors could differ [24]. For this reason, the F∣FortFoundation, a Dutch organization that is committed to improving the mental well-being of AYAs with cancer, organized a photovoice project for AYAs undergoing cancer treatment. They invited a group of Dutch AYAs to express themselves by taking photos of certain themes that are important to them together with a professional photographer.

In the present study, we aim to obtain a comprehensive view of the cancer experience of AYAs by using two visual methods: RPs and photovoice. All participants were asked to create an RP, and approximately half of the participants also took part in the photovoice project, in collaboration with the F|FortFoundation.

## Methods

### Study design

Two qualitative visual research methods were used: RP interviews and photovoice. The original project of the F|FortFoundation comprised the photovoice. We added RP interviews to be able to compare the methods and see if different themes would come up.

The present study adopted a constructivist research approach, acknowledging that knowledge is co-created between researchers and participants [26]. That is, the photos of the photovoice project are shaped by the participant, the photographer, and FvL (an experience expert). Additionally, the interpretation of the RP is the result of the interaction between participant and the interviewer ZB, a female PhD student with experience in doing RP interviews with oncology patients. To give an example: as ZB had experience with doing RP interviews with adult patients, she went in with knowledge of existing themes amongst adults, which could help her grasp the meaning of the RP better, but could also lead to her making presumptions. To minimize the latter in data analysis, we employed a second encoder, AK, who has a master’s degree in pedagogy and a PhD in child obesity. Additionally, EH, a female elderly care physician, with ample experience in RP research, was directly involved in data analysis.

### Photovoice project of the F|FortFoundation

#### Participants

AYAs (18–35 years old) who had cancer, or were in remission from cancer, could apply to the photovoice project by sending an introductory text about themselves to the F|FortFoundation. The project was promoted on social media, and on the website of the F|FortFoundation and partner organizations. There was no restriction in cancer type, demographic area within the Netherlands, or the illness period. In total, 35 AYAs applied to the photovoice project, of which six were male. Ages ranged from 22 to 36 years, and the most prevalent type of cancer was breast cancer (14 out of 35). From all 35 submissions, the second author FvL chose eleven participants, aiming for a diverse group, with different ages, cancer types, and cultural backgrounds. In this group of 11, four were male.

#### Data collection

To facilitate the photovoice, six relevant domains for AYAs with cancer were identified in a brainstorm session, organized by F|FortFoundation, in which FvL gave the main input. Defining six domains was to facilitate a structure where AYAs were asked to make one photo for each domain. The six domains were as follows: (1) the essence: “This is the pure definition of me”; (2) the wrong gear: “I am pushing the accelerator pedal, but I am not moving. I cannot keep up with my peers”; (3) concerns about the future: “What will happen with me after this treatment?”; (4) The remedy: “What I need to be able to handle the toughest moments of my treatment”; (5) Forms of interaction: “I have to take control over the interactions between me and others.”; and (6) The end: “What does death actually mean and how will dying look like for me?.”

All participants worked with a photographer for two days. A week before meeting the photographer, the participant was interviewed by FvL about how the six domains played a role in his/her life. After the interview, FvL, together with the photographer, developed a mood board based on the interview answers as preparation for the photography days. On the first photography day, FvL, the photographer, and the participant discussed the mood board and determined composition and location for the photos. The rest of the two days were used to make six photos, one for each domain. Photos could be portraits, still lives, landscapes, or any other composition. Each photo was accompanied by a caption, written by the F|FortFoundation and approved by the participants. F|FortFoundation developed a book with all the photos (ISBN: 978-90-9034928-2).

### RP interviews

#### Participants

F|FortFoundation informed the 11 photovoice participants about the complementary RP interview study and asked them to participate. The first author (ZB) contacted the seven consenting participants by phone to confirm their involvement. Subsequently, AYAs who indicated interest in the F|FortFoundation project, but were not chosen for the photovoice, where also invited by ZB to participate in the RP interviews.

#### Data collection

ZB conducted two RP interviews with each participant. For participants taking part in the F|FortFoundation project, the first interview was held before they started working with the professional photographer and the second interview was held around 2 to 3 months later, after completion of the photovoice project. For participants who were not involved in the F|FortFoundation project, we chose similar time intervals.

Participants were asked to make an RP about their experience of living with cancer, followed by a semi-structured interview. An example of a RP was shown to the participants to give them an idea of which icons and symbols they could use and how an RP might look when completed [19, 22]. When the RP was completed, the interviewer asked the participant to explain what she/he had drawn, starting with the open question, and then asked more specific questions about elements of the RP (e.g., about the colors, shapes, and specific elements). All RP interviews were audio recorded.

The first three interviews were held in a setting of participants’ preference: at their home or somewhere else, with or without someone else being present. However, due to the COVID-19 pandemic, we had to perform the remaining interviews online, using Microsoft Teams [27]. After the interviewer gave instructions on how to draw an RP, the participant was given approximately 30 min to complete the RP individually (with the video call muted). When the RP was completed, the participant would send a photo of his/her RP to the interviewer, who in turn, would share her screen with the photo so both participant and interviewer could look at the RP while discussing it.

### Data analysis

RPs and photos were first analyzed separately. RP Interviews were transcribed verbatim and were used to support the RP analysis. The combined RP/interview data was analyzed by ZB by applying analysis tables, in which the RPs and the corresponding interview text were grouped and analyzed using open coding, in an inductive approach, as described in detail before [19]. A second coder, AK, checked all codes and made some suggestions to rename and add some codes. After discussing this together, ZB agreed with these suggestions and no conflicts occurred. Subsequently, ZB clustered all codes into overarching (sub)themes.

We applied a similar approach to analyze the photos. Each photo was analyzed separately, by looking at what was in the photo and what was written in the caption. The first author, ZB, described what could be seen on the photo and then applied codes to the photo and the caption. After all photos were analyzed, the codes were clustered into subthemes and main themes. ZB discussed this process and the emerging themes with EH. Subsequently, ZB and EH compared the themes that emerged from the RPs to ones in the photos, explicitly looking for similar or different metaphors and visual motifs.

### Ethical considerations

No formal ethical approval was needed for the study, as confirmed by The Medical Ethics Review Committee of the Academic Medical Centre. Confidentiality of participants was ensured, and written informed consent was obtained from each participant.

## Results

### Participants

We interviewed twelve AYAs of whom seven participated in the photovoice. Eleven of the twelve AYAs were female, and the average age was 31 years (SD of 3.6 years). The AYAs had different types of cancer, including lymphoma, breast, ovarian, brain, or testicular cancer. Half of the AYAs received curative treatment, five received palliative treatment, and one did not answer that question. An overview of the demographics of the participants can be found in the Appendix.

### Findings

The two most predominant themes emerging from the data were struggles related to (a) the future and (b) defining one’s identity. These main themes were visible in the RPs as well as in the photovoice; however, slight differences in sub-themes were found. For instance, while in both RPs and photos the effects of the cancer and treatment on the body and body-image were mentioned, the uncertainty surrounding being able to become pregnant was only depicted in the RPs. Furthermore, the difficulty of dating was also depicted in both visual tools, but the impact of the cancer on experimenting with one’s sexual orientation was only visualized in the photovoice. We will expand on the two main themes with examples from the RPs and photos.

#### Concerns for the future

The AYAs expressed concerns for the future related to multiple areas of their life, like relationships, education, work, finances, and housing. Especially relationships, education, and work were often depicted and mentioned as areas in which they experienced significant struggles.

##### Relationships

**Fig. 1:**
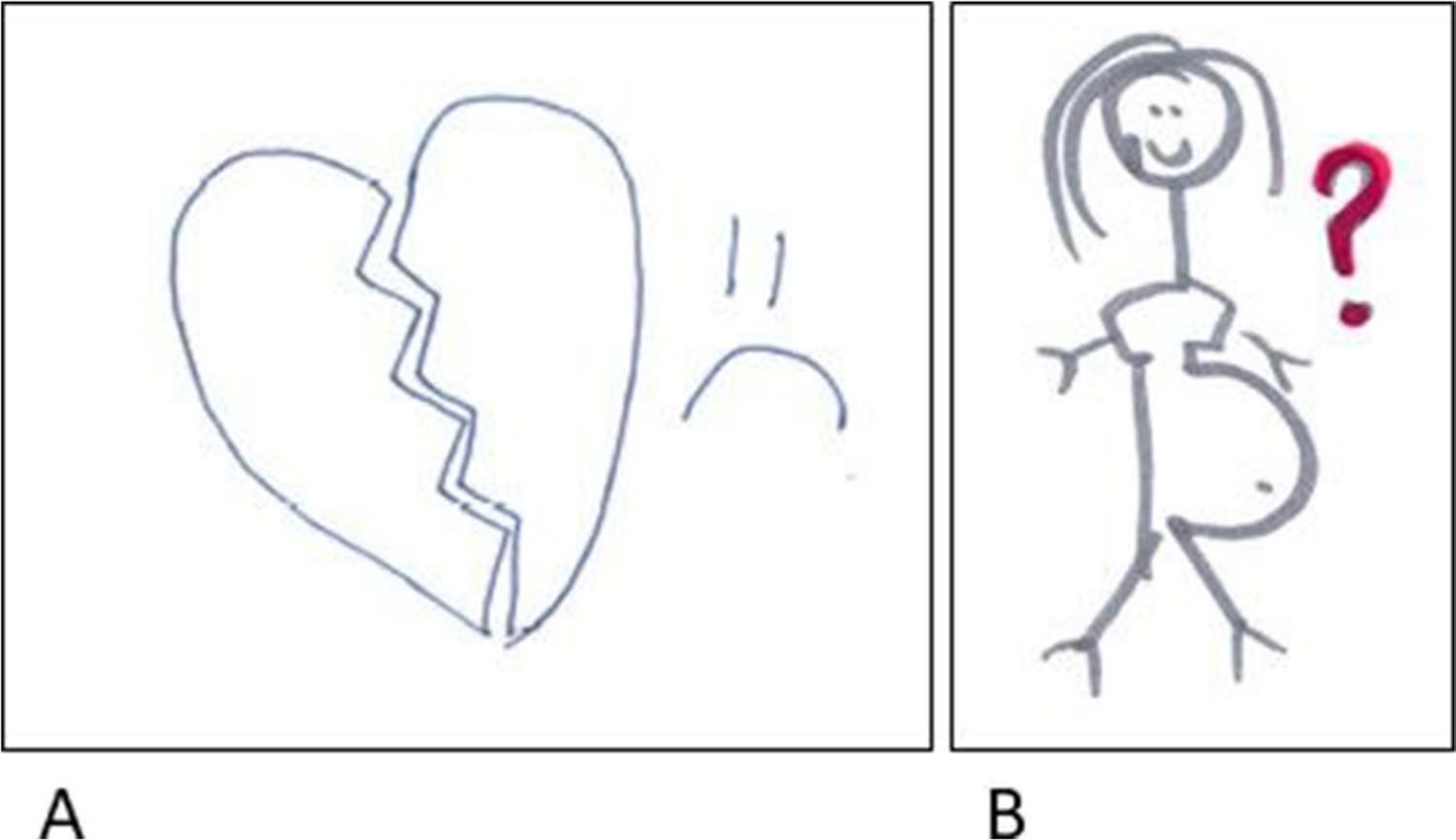
Relationships — **A** Part of RP of P7 that shows a broken heart and a sad looking face. **B** Part of first RP of P5 that shows herself with a pregnancy belly and with red marked question mark, depicting the uncertainty if getting pregnant is still possible

Participants raised multiple concerns regarding the impact of the cancer on relationships in their life. AYAs who did not have a partner, talked about the difficulty of dating during cancer treatment and were worried about how they would be able to find a love partner even after (complete) remission. For instance, a 28-year-old female with lymphoma drew a big broken heart with a sad face next to it on her RP (Figure 1a). She explained that she tried to go on dates during her treatment, but had difficulties with the fact that she did not want to tell everyone that she had cancer. She said: “I am of course not the same person I was before I got sick. So that also makes it very hard to get to know new people, because, will you tell the person this and if so, when will you tell that person?” Two other AYAs, a 31- and a 32-year-old female depicted their wish to have another child and talked about the insecurity whether becoming pregnant was still possible due to the chemotherapy. Figure 1b shows an example of how this was depicted in an RP.

##### Education and work

**Fig. 2:**
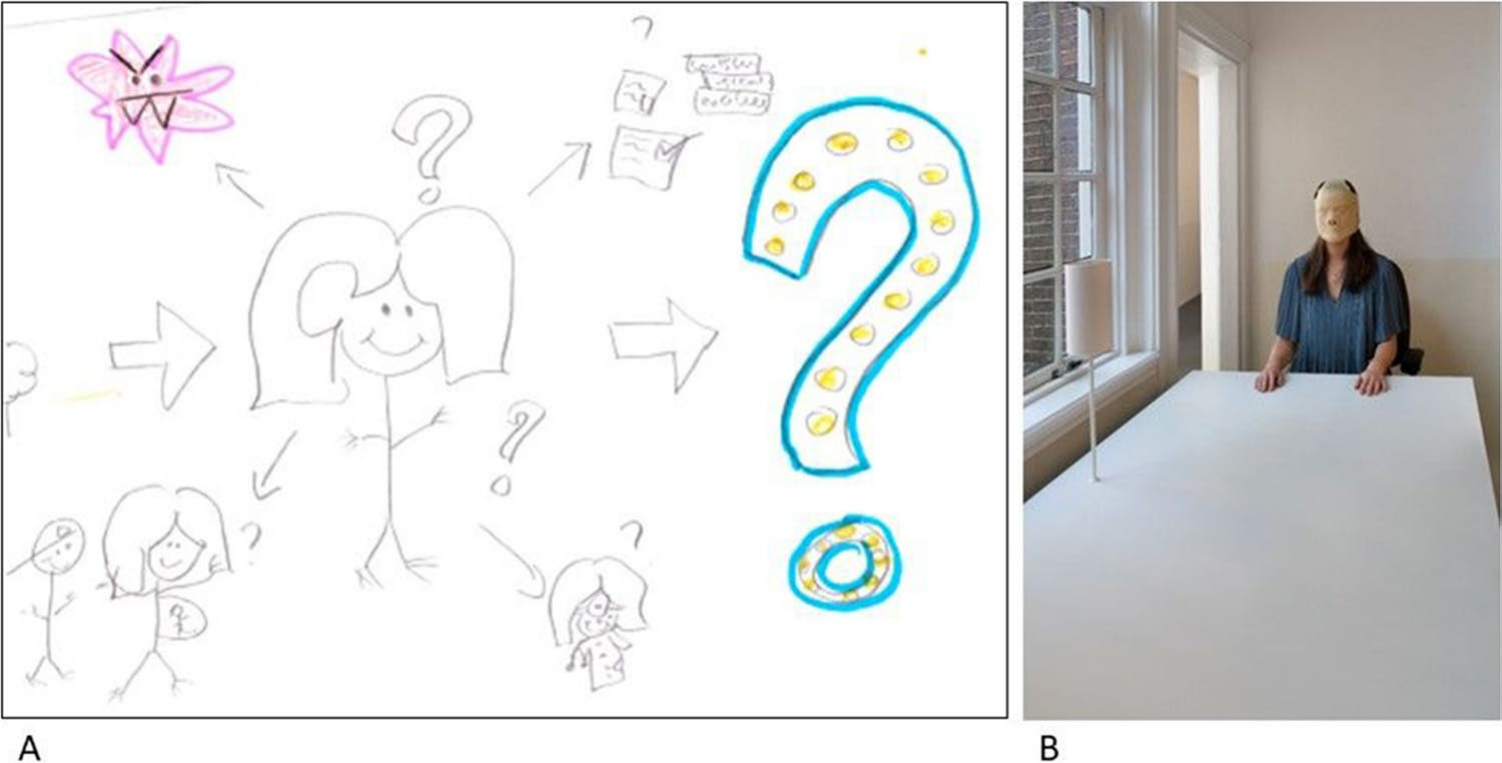
Education and work — **A** Part of first RP of P2 in which she drew herself surrounded by elements that are uncertain in the future, including the tumor, education, getting a career, and getting pregnant. **B** Photo from photovoice project in which the AYA is wearing her radiation mask to a job interview

A 23-year-old female expressed multiple concerns regarding her future. In her RP, she drew herself surrounded by all her concerns for the future (Fig. 2a). She drew her tumor, her education, work, and herself being pregnant. Her concerns surrounding work was also the topic of one of her photos. On the photograph, she sits at a white table with the mask of the radiation therapy on her face (Fig. 2b). The photo is accompanied by the caption: “At job interviews they will come up with excuses for not hiring me. I already want to shout it out: Look at me, look at my skills, and do not look for traces of my disease.”

#### Struggles with (re)defining one’s identity

The cancer obstructs the identity of AYAs in many ways. For instance, many AYAs expressed that they felt segregated from their peers and being perceived differently. The cancer also exerted an effect on their body- and self-image, and on exploring one’s sexual orientation, which may induce a need to redefine their identity.

##### Feeling segregated from peers

**Fig. 3:**
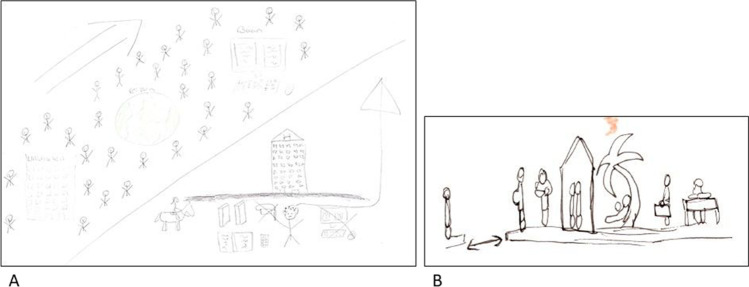
Feeling segregated from peers — **A** The first RP of P3 in which the AYA drew a line between her and her peers, depicting how she felt completely segregated from her peers. **B** Part of first RP of P9 where she drew herself on the island on the left, and others her age on the island on the right accomplishing things she cannot do because of the cancer

A 25-year-old female focused her first RP on feeling segregated from her peers and her life coming to a standstill, while others of her age were developing careers and enjoying life (Fig. 3a). She literally drew a line separating herself from her peers. Another example is a 32-year-old female who drew two islands in one of her RPs: one with herself on it and the other island inhabiting other people (Fig. 3b). Other people get pregnant, move in together, go on holidays, and get jobs. From her own island, she is looking at them with envy, not being able to have this herself.

##### Perceptions of others

Many AYAs talked about their concerns of how others were perceiving them. For instance, they suddenly became an ill person, as if that was now their sole identity. One AYA (28-year-old female) said: “Suddenly you are that sick girl.”

**Fig. 4:**
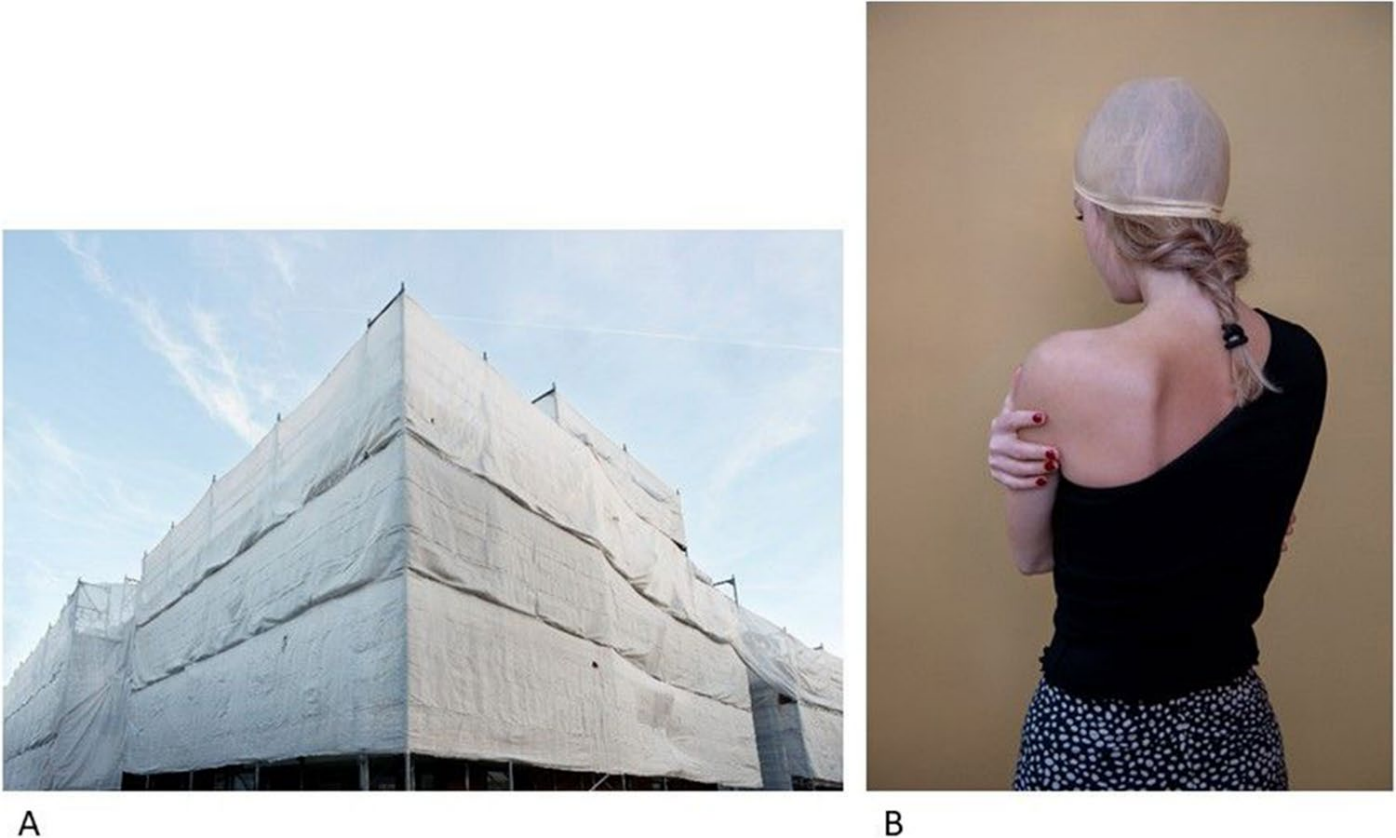
Perceptions of others — **A** Photo from photovoice project of a building with scaffolds in opaque covering, to depict that no one can see the progress that is made inside. **B** Photo from photovoice of an AYA with a hairnet on, her back to the camera, and holding herself

A 25-year-old female was afraid others thought she was lazy. A photo from the photovoice shows a building with scaffolds in opaque covering (Fig. 4a) — a metaphor for her working on her personal development, but people not being able to see this from the outside. A second photo of this participant pictures herself with a hairnet on, her back to the camera, and holding herself (Fig. 4b), accompanied by the following text: “You can’t see anything from the outside. That’s why they often cannot believe it, cannot comprehend it. […] Sometimes I fantasize about being bald. Would people then realize that this is real? (But thank god I still have my hair).”

##### Changing appearance and self-image

**Fig. 5:**
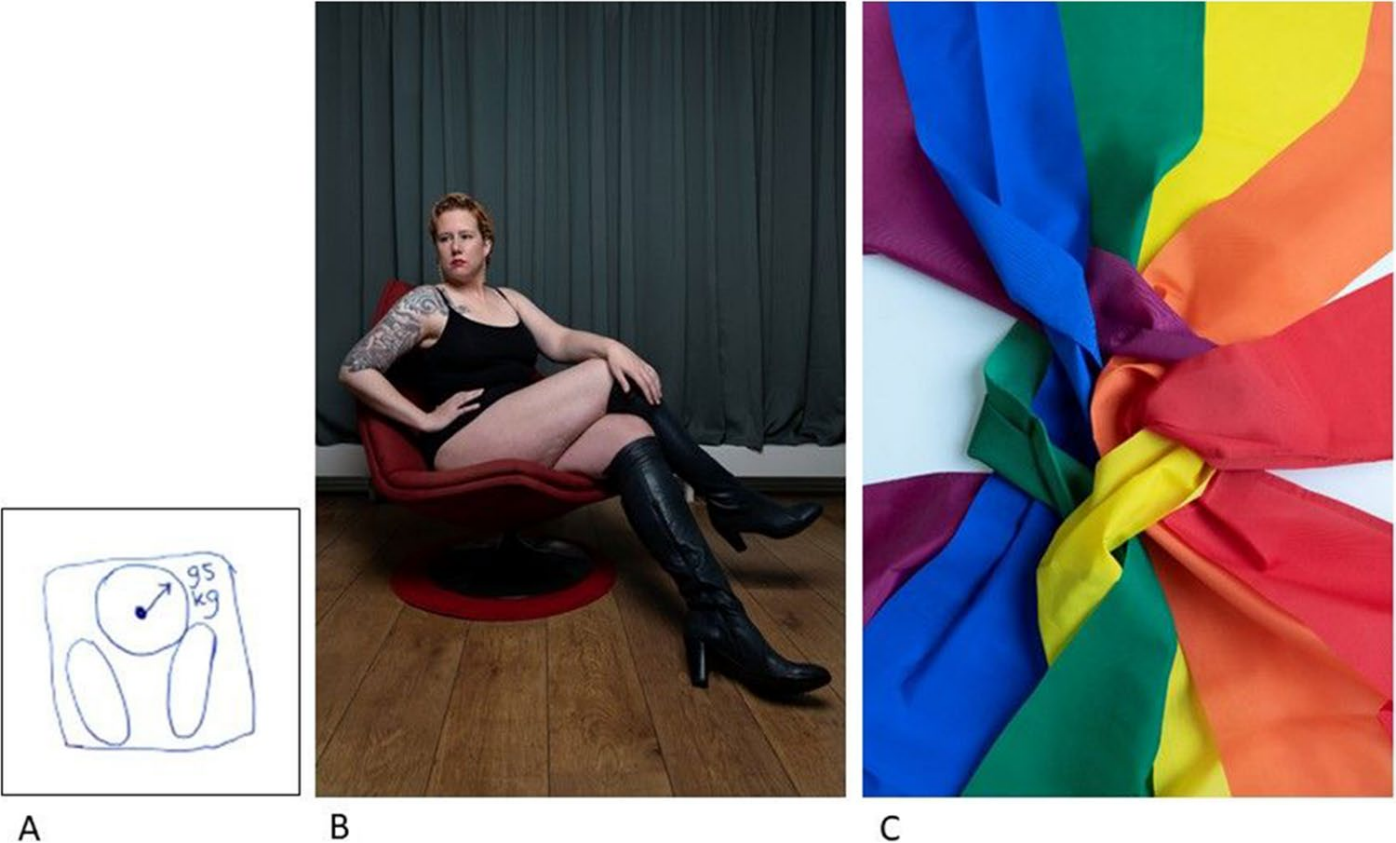
Changing appearance, self-image, and sexual orientation — **A** Part of RP of P7 that shows a scale, depicting how she gaining weight during cancer treatment. **B** Photo from photovoice project of an AYA showing off her changed body, that she trouble accepting. **C** Photo from photovoice project of entangled ribbons in the colors of the rainbow, referring to the LGBTQ community

Multiple AYAs talked about their appearance changing. A 28- and a 32-year-old female talked about how chemotherapy made them lose their hair and gain weight. Regarding her weight, the 28-year-old drew a scale in the RPs (Fig. 5a): “I drew a scale, because I really gained a lot of weight during the chemotherapy, ten kilos. Well, I was not happy about that. […] Weight also really is a thing that I of course think about a lot.” The other AYA had a photo made in which she is sitting in a chair with just a tank top and big boots on, showing part of her body (Fig. 5b). The caption of the photo shows that she had a hard time accepting her changed body: “Hair loss, amputation, weight gain; I am trying to accept myself and I know I can be proud, but it often does not feel like that. I worked hard to love my body, to form it to my image of a hot woman. Now I lost all footing.”

##### Sexual orientation

One of the participants (32-year-old female) had a photo made to express her struggles with experimenting with her sexual orientation during the cancer treatment (Fig. 5c). The photo shows ribbons in the colors of the rainbow that are tangled, and is accompanied by the caption: “I was ready for it, creating an account on Tinder, to figure out if I like women or men, maybe something in between. Now it’s way too complicated. Do I use an old photo or a photo with me without hair? The fear of rejection paralyzes me.”

## Discussion

We aimed to obtain a comprehensive view of the cancer experience of AYAs by using the visual methods RPs and photovoice. It became clear that cancer has an effect on many aspects of an AYA’s life and impacts their development in areas such as relationships, fertility, education, employment, self-image, and sexual orientation.

In previous studies about experiences amongst AYAs with cancer, especially concerns regarding education and employment were mentioned frequently. Literature reviews from Warner et al. [1] and Fardell et al. [2] reported several studies on the negative impact of cancer on educational trajectories and employment of AYAs. Our data provide further support for these experiences of AYAs. What also becomes apparent from the extant literature is that most studies on cancer experiences of AYAs are quantitative [1, 2, 28, 29]. Our qualitative study adds to current literature by providing visuals to accompany written and spoken text. RPs and photovoice are able to present the story of an AYA in one image (RPs) or a collection of images (photovoice), instead of providing large amounts of text. Furthermore, the fact that participants have control over the topics they want to depict helps with determining which themes are most important to them.

To the best of our knowledge, RPs, or other drawing methods, have not been used to explore experiences of AYAs with cancer before. Drawing as a method to gain insight into experiences has mostly been used among children and older adults [13–17, 30, 31]. Photovoice has been conducted with AYAs, but only with cancer survivors, not AYAs currently undergoing cancer treatment [24, 32, 33]. Studies from Yi [32, 33] have described late medical and psychosocial effects of surviving (childhood) cancer and retrospect experiences of the treatment period. Our findings focused on present experiences with cancer treatment, which gave us valuable information on which themes play a role during treatment, such as being forced to put a halt to studying and/or working, feeling segregated from peers, experiencing changes in appearance, and struggles surrounding dating during treatment. Besides, our study proves that AYAs under treatment are able to participate in photovoice — although time and energy-consuming - and all AYAs described their participation as a very valuable experience. Hence, we found that RPs and photovoice have the potential to uncover experiences of AYAs currently undergoing cancer treatment.

Using two visual tools allowed us to compare specific affordances of the two tools. We were able to see substantial overlap in themes, but also found that the methods could complement each other. In some cases, the RP gave a deeper insight into what a theme meant to an AYA, while in other cases, the photos provided a stronger view. These disparities could stem from the fact that the photovoice started with predetermined domains, while this was not the cause with the RPs. Furthermore, the photos were made in consensus between photographer, patient, and FvL, contrary to the RPs were patient had full control over what they wanted to depict.

Comparing the findings of the present study to the RP study we previously conducted with adults, we see that main themes are similar [19]. However, the AYAs placed more emphasis on the effect of the cancer on defining their identity. For example, topics as (online) dating, pregnancies, and problems with studying or finding a career path, were very important to AYAs, but hardly mentioned by the adults in our previous study [19]. Hence, our findings add to the notion that AYAs represent a unique cancer population [1–4].

Furthermore, our findings imply having cancer at such a young age can be extremely difficult. Based on our results as well as other [1–4, 28, 29], we argue that AYAs are in need of targeted interventions, especially focusing on improving psychosocial health. For instance, more initiatives such as the AYA care team from the “National AYA Young and Cancer Care network,” a Dutch organization, could be developed. The AYA care team is active in every academic hospital in the Netherlands and assigns every AYA with cancer an AYA nurse, a medical psychologist, a sexologist, an occupational physician, a fertility specialist, and a social worker.

### Strengths and limitations

Three strengths of this study are noteworthy. First, all phases of data analysis were executed by at least two authors, which stimulated dialogue between authors in order to prevent tunnel vision [34]. Second, the involvement of FvL, an experience expert, as interviewer at the start of the photovoice, allowed participants to feel understood and more comfortable to tell their story. Third, the fact that the interviewer of RP interviews, ZB, was a young adult herself and thus was in the same life phase as the AYAs, could also have increased connection and understanding between AYAs and interviewer.

A limitation of the study is that the photovoice started with a priori themes which were not used in the RPs. The RPs were also not taking into account at the time of deciding on the a priori themes in the photovoice. Photovoice and RPs were treated as two completely separate elements, while integrating them might have been even more beneficial for gaining insight into the experiences of the AYAs. Another limitation is that only one male participated in our study, possibly making our findings less representative to the general AYA generation. Lastly, the methods could be very demanding for participants and are therefore possibly difficult to use for AYAs with advanced stages of cancer.

## Conclusion

Our visual tools show a detailed picture of what it is like to have cancer as an AYA. We found that cancer has an effect on many aspects of an AYA’s life, such as relationships, fertility, education, employment, and self-image. Further research on how these themes play a role in the life of AYAs with cancer is needed.

## Appendix

**Table 1.** Demographics of the participating AYAs

Participant number	Gender	Age	Type of cancer	Time between diagnosis and first interview (in months)	Treatment	Participant in F|FortFoundation project
*P1*	Male	35	Testicular cancer	5	Curative	Yes
*P2*	Female	23	Brain cancer	5	Palliative	Yes
*P3*	Female	25	Ovarian cancer	16	Palliative	Yes
*P4*	Female	34	Breast cancer	16	Curative	Yes
*P5*	Female	32	Lymphoma	11	Curative	Yes
*P6*	Female	32	Breast cancer	64	Curative	No
*P7*	Female	28	Lymphoma	16	Curative	Yes
*P8*	Female	33	Breast cancer	45	Palliative	No
*P9*	Female	32	Breast cancer	8	Curative	Yes
*P10*	Female	30	Breast cancer	22	Palliative	No
*P11*	Female	35	Breast cancer	14	Missing	No
*P12*	Female	31	Lymphoma	19	Palliative	No

Table 1
